# Association of immune evasion in myeloid sarcomas with disease manifestation and patients’ survival

**DOI:** 10.3389/fimmu.2024.1396187

**Published:** 2024-08-07

**Authors:** Marcus Bauer, Astrid Monecke, Hubert Hackl, Andreas Wilfer, Nadja Jaekel, Hendrik Bläker, Haifa Kathrin Al-Ali, Barbara Seliger, Claudia Wickenhauser

**Affiliations:** ^1^ Institute of Pathology, Martin Luther University Halle-Wittenberg, Halle, Germany; ^2^ Institute of Pathology, University Leipzig, Leipzig, Germany; ^3^ Institute of Bioinformatics, Biocenter, Medical University Innsbruck, Innsbruck, Austria; ^4^ Krukenberg Cancer Center Halle, University Hospital Halle, Martin Luther University Halle-Wittenberg, Halle, Germany; ^5^ Department of Hematology, University Hospital Halle, Martin Luther University Halle-Wittenberg, Halle, Germany; ^6^ Medical Faculty, Martin Luther University Halle-Wittenberg, Halle, Germany; ^7^ Fraunhofer Institute for Cell Therapy and Immunology, Leipzig, Germany; ^8^ Institute of Translational Immunology, Medical School “Theodor Fontane”, Brandenburg an der Havel, Germany

**Keywords:** myeloid sarcoma (MS), immune evasion, HLA, survival, TME (tumor microenvironment)

## Abstract

**Introduction:**

Myeloid sarcomas (MS) comprise rare extramedullary manifestations of myeloid neoplasms with poor patients’ outcome. While the clinical relevance of the tumor microenvironment (TME) is well established in many malignancies, there exists limited information in MS.

**Methods:**

The expression of the human leukocyte antigen class I (HLA-I) antigens, HLA-I antigen processing and presenting machinery (APM) components and the composition of the TME of 45 MS and paired bone marrow (BM) samples from two independent cohorts were assessed by immunohistochemistry, multispectral imaging, and RNA sequencing (RNAseq).

**Results:**

A significant downregulation of the HLA-I heavy chain (HC; 67.5%) and ß2-microglobulin (ß2M; 64.8%), but an upregulation of HLA-G was found in MS compared to BM samples, which was confirmed in a publicly available dataset. Moreover, MS tumors showed a predominantly immune cell excluded TME with decreased numbers of tissue infiltrating lymphocytes (TILs) (9.5%) compared to paired BM (22.9%). RNAseq analysis of a subset of 10 MS patients with preserved and reduced HLA-I HC expression revealed 150 differentially expressed genes and a significantly reduced expression of inflammatory response genes was found in samples with preserved HLA-I expression. Furthermore, low HLA-I expression and low TIL numbers in the TME of MS cases were linked to an inferior patients’ outcome.

**Discussion:**

This study demonstrated a high prevalence of immune escape strategies in the pathogenesis and extramedullary spread of MS, which was also found in patients without evidence of any BM pathology, which yields the rational for the development of novel individually tailored therapies for MS patients.

## Graphical Abstract

Tumor samples of 45 patients from two centers with a diagnosis of myeloid sarcoma (MS) and paired bone marrow biopsies (BMBs) were stained for components of the human leukocyte antigen class I (HLA-I), antigen processing and presenting machinery (APM) and tissue infiltrating lymphocytes (TILs) using conventional immunohistochemistry (IHC) and multispectral imaging (MSI). The publicly available RNA expression dataset GSE103344 originating from human AML cells with the *in vitro* and *in vivo* ability to form solid tumor mass served as an external reference for APM component expression. In addition, bulk RNA sequence analysis was performed from MS samples with preserved and decreased HLA-I expression. Higher APM expression and TIL infiltration was correlated with a superior survival of MS patients.

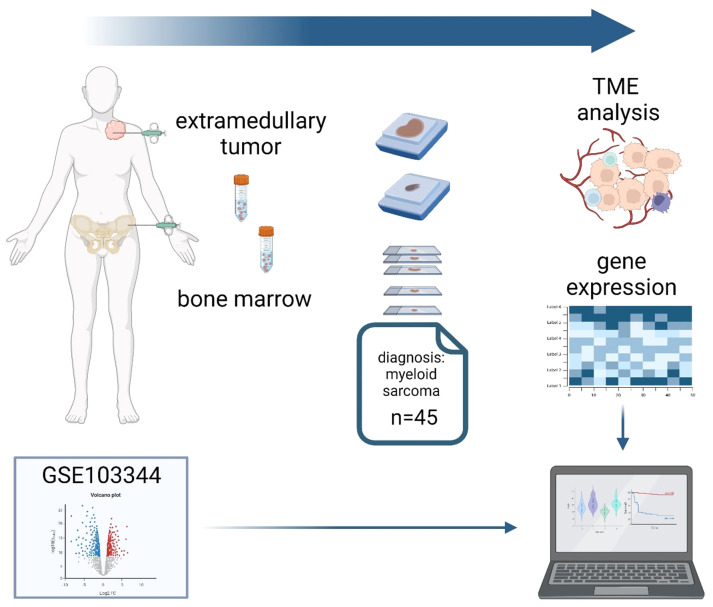

## Introduction

Myeloid sarcomas (MS) encompass a heterogeneous group of tumor mass forming clonal hematologic diseases with an extramedullary manifestation that is usually associated with poor patients’ outcome. Frequently skin, lymph nodes, gastrointestinal tract, bone, soft tissue, central nervous system and testes are affected. MS can develop in context with an acute myeloid leukemia (AML), myeloproliferative neoplasm (MPN), myelodysplastic neoplasm (MDS) or at relapse, particularly following allogeneic hematopoietic stem cell transplantation (1–4). Indeed, about 70% of patients exhibit concordant molecular alterations in both MS and bone marrow (BM) disease implying a potential origin from a shared hematopoietic stem cell or precursor (5, 6), although approximately 25% of the disease occurs without BM involvement (4, 7). Furthermore, prevalence for males over females and a mean onset in the 4^th^ and 5^th^ decade has been shown (4, 8). The outcome of the vast majority of patients is poor and an influence of the underlying myeloid neoplasm (MN) has been controversially discussed (1, 8, 9). Due to its rarity and lack of randomized controlled studies, the diagnosis of MS is challenging and might result in misdiagnosis as lymphoma (7, 8). MS express myeloid markers like myeloperoxidase, lysozyme, CD33 or CD68, as well as T cell surface markers, such as CD3, CD4 and/or CD5, but frequently negative for immature markers like CD34 (10, 11). Recently, various genetic aberrations have been identified in MS samples (12), which were also of prognostic relevance (13). Radiotherapy, surgery and allogeneic stem cell transplantation are currently used for the treatment of MS patients (14). Furthermore, low-dose therapy with hypomethylating agents (HMA) after stem cell transplantation has shown an improved overall survival (OS) due to the activation of an anti-tumor immune response (14), while recent advances in genetic profiling of MS samples may also enable the implementation of targeted therapies in these patients (15).

Next to genetic abnormalities, different immune escape mechanisms including an altered expression of HLA-I HC and ß_2_M, soluble and metabolic factors as well as an increased expression of inhibitory immune checkpoint (ICP) molecules have been identified in myeloid malignancies, which also might play an important role in MS (16–18). In order to uncover the role of the immune evasion strategies in MS pathophysiology, this study analyzed the tumor microenvironment (TME) with special focus on the composition and function of the immune cell infiltrate as well as the expression of immune-relevant markers in MS and/or paired BM samples. In addition, 10 selected MS cases with distinct HLA-I expression levels were subjected to RNAseq analysis.

## Materials and methods

### Patient samples and ethics approval

Formalin-fixed and paraffin-embedded (FFPE) MS samples and bone marrow biopsies (BMB) (n=83) from 45 patients (38 MS samples with paired BMBs and further 7 MS cases without paired BMB) were collected in the period from 2011 to 2022 and archived at the Institutes of Pathology of the Martin-Luther University Halle-Wittenberg, Germany (n=29) and the University of Leipzig, Germany (n=16), respectively. The use of the FFPE BMB was approved by the Ethical Committee of the Medical Faculty in Halle, Germany (2017-81 and 2023-196). Clinical data from these patients were available, such as age, gender, disease status, therapy and survival time (see **Table 1**).

**Table 1:** Clinicopathological characteristics of patients.

variable		value
**age**	[mean] years	20-79 [54]
**sex**	male/female (n=45)	25/20
**underlying**	AML (n)	24
** BM finding**	MDS & MDS/MPN (n)	5
	MPN (n)	11
	non-neoplastic BM (n)	5
**follow-up**	available number of patients (n)	24
	survival time [mean] (months)	1-25.1 [7.8]

### Standard morphological evaluation of the bone marrow and immunohistochemistry

Histopathological diagnostics were performed according to the diagnostic criteria of the World Health Organization (WHO) classification of Tumors of Hematopoietic and Lymphoid tissues, fourth edition 2017 and 2022 (3, 4, 19). Conventional histopathology of the MS cases was performed employing H&E staining and chloroacetate esterase reaction. Immunohistochemistry (IHC) was performed on all samples using antibodies (Ab) directed against CD33, CD34, CD117, MPO, lysozyme, CD68, HLA-I HC, ß_2_M, tapasin (tpn), TAP1, TAP2 and HLA-G according to the suppliers’ instructions. The Ab are summarized in **Supplementary Table S1**. For the expression analysis of HLA-I HC, ß_2_M, tapasin (tpn), TAP1, TAP2 and HLA-G, the H score was employed as described elsewhere (20). A high expression of the respective proteins refers to a H score >150.

### Analysis of APM genes using publicly available RNA data

In order to compare the APM component expression in human AML cells that showed mass formation *in vitro* and *in vivo*, a publicly available dataset (GSE103344) (21) containing Affymetrix Human Gene 2.0 ST mRNA Array data of human THP-1 AML cells with knock down of RKIP that showed a role in tumor mass formation *in vitro* and *in vivo* (21). The differentially gene expression (DGE) of various APM components was analyzed using the Gene Expression Omnibus (GEO) repository (https://www.ncbi.nlm.nih.gov/geo/). Differentially expressed genes (DEG) of AML cells with knock down of RKIP and respective controls with preserved RKIP were analyzed and visualized using the GEO2R tool (https://www.ncbi.nlm.nih.gov/geo/geo2r/).

### Multispectral imaging

Multispectral imaging (MSI) was performed as recently described (22) employing a six-plex Ab panel with CD3, CD8, FoxP3, MUM1p, CD34, and granzyme B (GrB). Briefly, after antigen retrieval at pH 6 or 9 depending on the Ab used, the tissues were incubated for 30 min with the primary Ab followed by the secondary Ab (Akoya biosciences, Marlborough, MA, USA, Opal Polymer HRP Ms + Rb) for 10 min. Tyramide signal amplification (TSA) visualization was performed using the Opal seven-color IHC kit containing the fluorophores Opal 520, Opal 540, Opal 570, Opal 620, Opal 650, Opal 690 (Akoya biosciences, Marlborough, MA, USA) and DAPI. Stained slides were imaged employing the Pheno Imager HT (Akoya biosciences, USA). Cell segmentation and phenotyping of cell subpopulations were performed using the inForm software (Akoya biosciences, USA). The frequency of immune cell populations and their cartographic coordinates were evaluated for immune cell enumeration and relationship analysis using the R scripts from the phenoptr and phenoptrReports packages (https://github.com/akoyabio). Moreover, all CD3 stains of the MS samples were analyzed by a pathologist and the immune cell infiltration pattern was grouped in “immune cell excluded” tumors with immune cells located at the margin of the tumor and “immune cell infiltrated” tumors with a diffuse immune cell infiltration into the tumor (23).

### RNA isolation, RNA sequencing and data analysis

Four to five 10 µm thick FFPE tissue slides were prepared. Total RNA was isolated with Maxwell RSC RNA FFPE Kit (Promega, USA) according to the manufacturer’s instructions. For RNA sequencing (RNAseq), 2 μg of total RNA/sample was employed and strand specific 150 bases paired-end RNAseq was done using the Illumina NovaSeq platform by Genewiz (Leipzig, Germany). Approximately 20 million reads per sample were obtained. Reads were trimmed using Trimmomatic, quality checked using fastqc 0.11.9 and mapped to the human reference genome (hg38) using splice aware aligner STAR 2.7.9a. Quantifications on NCBI gene models (hg38refGene) were performed using featureCounts v2.0.0. Differential gene expression analyses between HLA-I HC^high^ versus HLA-I HC^low^ samples was performed based on a negative binomial distribution using the R package *DESeq2* (24). P-values were adjusted based on the false discovery rate (FDR) according to the Benjamini-Hochberg method. Genes with an average expression across all samples (base mean) >10, more then two-fold change, and a FDR<0.1 were considered as significantly differentially expressed and visualized in a volcano plot using the R package *EnhancedVolcano*. Gene set enrichment analysis was performed on log2-fold changes with the GSEA tool v4.2.3 (25) using hallmark genesets (MSigDB) and results were visualized as bubble plots.

### Statistics

The Mann–Whitney U test was employed to compare clinical data. Patients with missing information in any other variable were excluded from regression analyses. Cox regression analyses were performed using IBM SPSS. Kolmogorov–Smirnov test revealed non-parametric data (p < 0.05). The Mann–Whitney U test was used to compare clinical data, frequencies of immune cell subpopulations and the expression pattern of immune-relevant markers. Survival analyses were performed on 24 patients (follow-up time of 25 months) using the Kaplan-Meier estimators and differences calculated with log-rank tests or Cox regression models. P values < 0.05 were considered statistically significant. The figures were generated using the GraphPad Prism 7.0 software, IBM SPSS Statistics 28.0 and biorender (https://www.biorender.com).

## Results

### High prevalence of low HLA-I APM component and high HLA-G expression in MS

**Figure 1:**
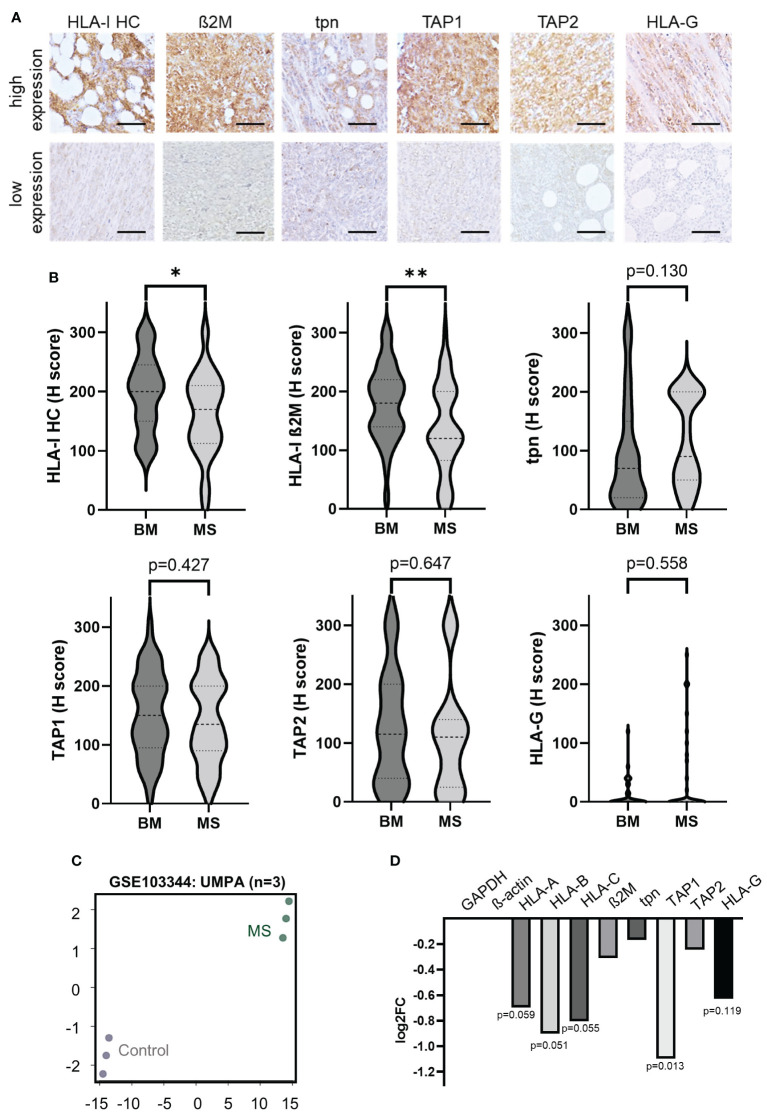
Expression of HLA-I antigen processing and presenting machinery (APM) components and non-classical HLA-G in myeloid sarcoma patients. **(A)** Representative IHC stainings of HLA- I HC, ß_2_M, tpn, TAP 1 and 2 and HLA-G with high and low expression levels, respectively. All IHC stainings were analyzed employing the H score as described in Materials and Methods. The scale bars depict 50 µm. **(B)** Results are summarized in Violin plots and their significance is shown in p-values (* p<0.05; ** p<0.005). **(C)** RNA expression data of publicly available GSE103344 data set were analyzed and principal component analysis (PCA) is shown (controls n=3 [unmodified THP-1 AML cells] and MS formations n=3 [THP-1 AML cells with knock down of RKIP]). **(D)** Results of differential gene expression (DGE) analysis is shown in a bar graph showing the log2 fold change of different APM components The p-values are given underneath the bars.

In order to determine the expression levels of HLA-I APM components in MS, protein expression of HLA-I HC, ß_2_M, TAP1, TAP2 and tpn was determined by conventional IHC (**Figures 1A, B**). Low HLA-I HC and β_2_m expression levels (H-score <150) were found in 50.0% (19/38 MS cases) and 63% (28/45 MS cases), respectively. Tpn expression was low in 57.7% (26/45 cases), TAP1 and/or 2 in 62.2% of MS samples (28/45 cases), respectively. In general, 37/45 MS cases (82.2%) showed an impaired expression of at least one APM component. As an alternative immune escape mechanism a high HLA-G expression was found in 12/44 cases (27.3%), which was accompanied in 9/12 (75.0%) cases by concordant high HLA-I HC expression levels with an H-score >150. No significant differences in results were detected by comparing tissue specimens of the two different pathology departments (**Supplementary Table S2**). In addition, a publicly available dataset (GSE103344) of RNA expression analysis of human AML cell lines was evaluated for DGE. As shown in **Figures 1C, D**, a downregulation of HLA-I APM components with particularly decreased mRNA expression of the HLA-I HC and TAP1 was detected.

### Comparison of the individual HLA-I APM component expression in BM and corresponding MS

**Figure 2:**
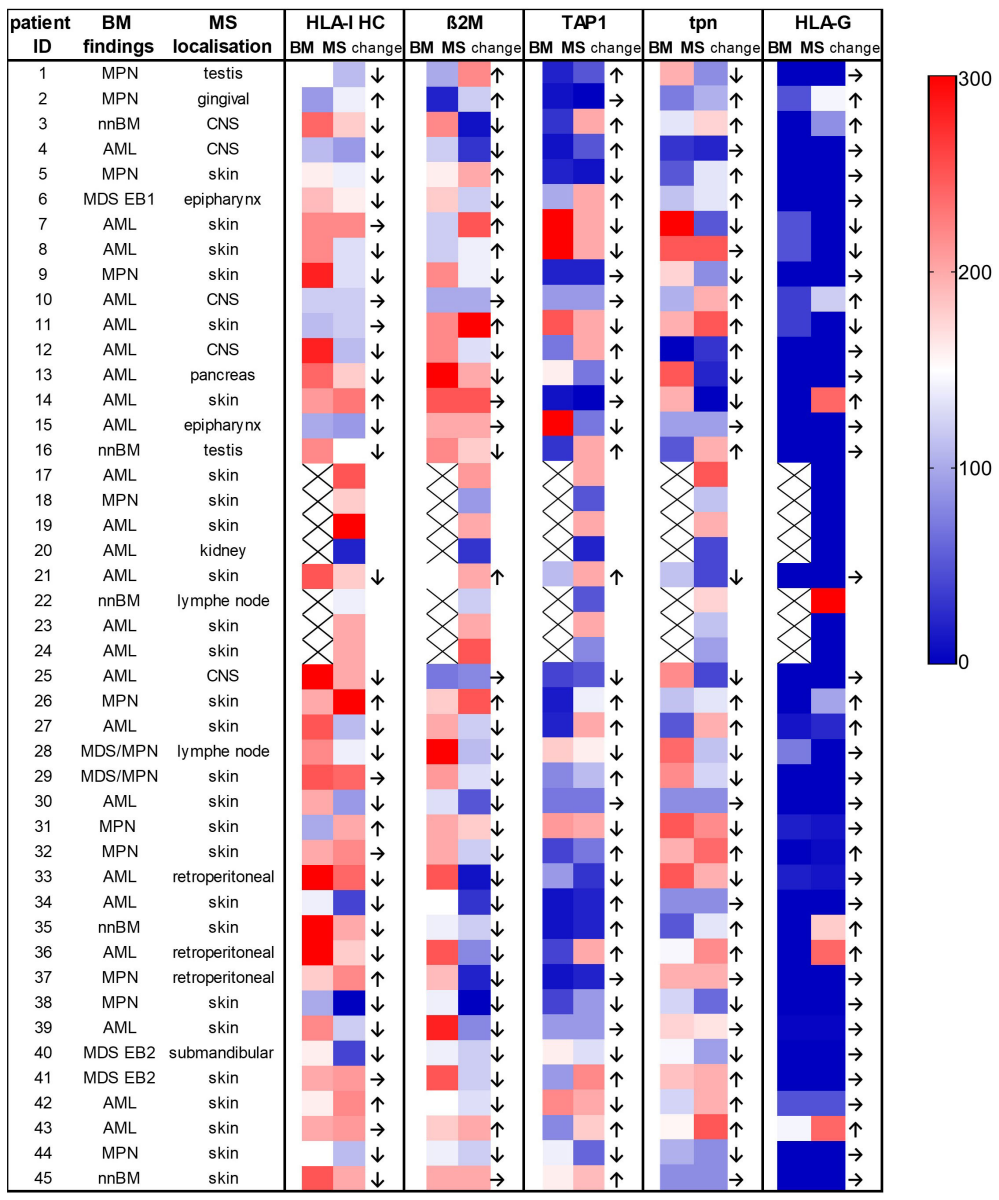
Comparison of the expression of the HLA-I antigen processing and presenting machinery (APM) components in individual patients with paired bone marrow and myeloid sarcoma samples. The localization of the MS and the underlying BM findings including different myeloid neoplasia are provided on the left side of the figure. The expression levels of the different proteins analyzed by conventional IHC are shown as H scores. Higher values are shown in red, lower values in dark blue (see the color legend at the right side). MS showed a downregulation of HLA-I HC in 67.5% and of ß2M in 64.8% of cases when compared to BM samples. nnBM, non-neoplastic bone marrow.

To elucidate the immune escape mechanisms within the evolution of MS in an individual patient, the HLA-I APM component expression was analyzed between matched BMB and MS tumors available from 38/45 MS cases (84.4%) (**Figure 2**; **Table 2**). In 4 cases, no neoplasia in the bone marrow could be detected. A downregulation of HLA-I HC and ß_2_M expression was found in 65.7% (25/38) and 63.1% (24/38) of MS tumor cases, respectively, while in 4 patients a higher HLA-I HC expression was detected in the MS vs. BMB. A downregulation of TAP1 was found in 40.5% of MS cases compared to corresponding BMB, while tpn expression was only downregulated in 27.1%, but upregulated in 29.7% of MS cases. Furthermore, HLA-G expression was higher in 27.0% of MS cases, but lower in 8.1% compared to BMB. No significant differences in the HLA-I APM component expression was found regarding the underlying diseases or the anatomical localization (**Supplementary Tables S3**, **S4**).

**Table 2:** Composition of the TME and immune-relevant markers.

variable	bone marrow	myeloid sarcoma	*x* ^2^ p-value
HLA^-^ I HC mean (range)	197 (90-300)	161 (0-300)	0.335
ß_2_M mean (range)	180 (20-300)	135 (0-300)	0.189
tpn mean (range)	93 (10-300)	115 (0-300)	0.012
TAP1 mean (range)	151 (10-300)	139 (10-250)	0.338
TAP2 mean (range)	125 (10-300)	113 (0-300)	0.596
HLA-G mean (range)	12 (0-120)	32 (0-300)	0.205
TIL mean (range)	22.9 (0.9-54.1)	9.57 (0.2-40.3)	0.413
T cells mean (range)	9.16 (0.2-46.1)	3.93 (0.1-20.3)	0.431
CD8^+^ T cells mean (range)	1.85 (0.0-18.6)	0.54 (0.0-4.6)	0.235
GrB^+^ cells mean (range)	3.59 (0.0-18.7)	5.91 (0.0-6.2)	0.175
Treg mean (range)	0.36 (0.0-6.3)	0.57 (0.0-10.7)	0.452
MUM1p^+^ cells mean (range)	1.05 (0.0-6.7)	1.10 (0.0-9.8)	0.246

### Characterization of the TME in MS and its link to HLA-I APM component expression

**Figure 3:**
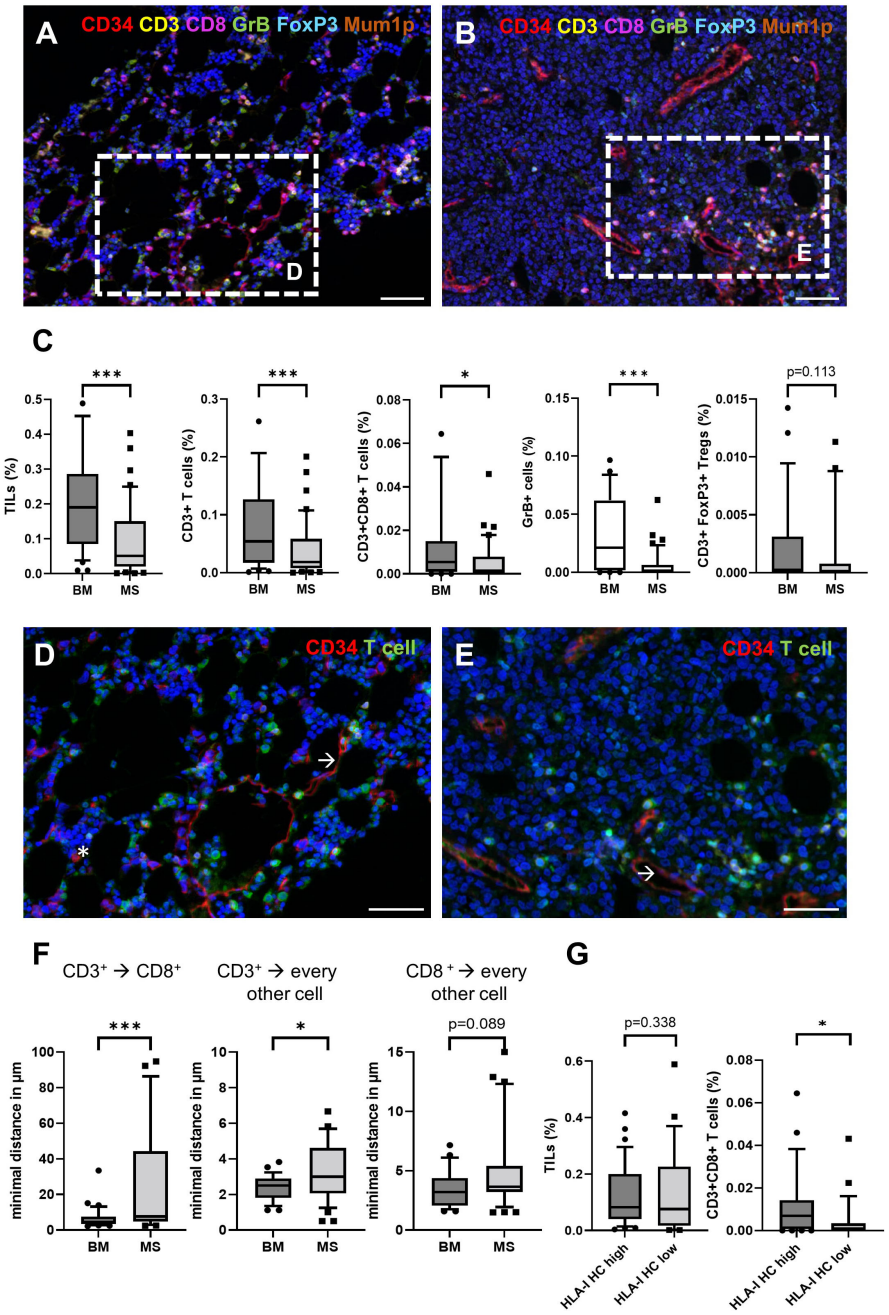
Comparison of the frequency and spatial distribution of the cellular immune subpopulations in the TME of BM and MS cases. Paired BM **(A)** and MS samples **(B)** showed a decreased density of CD3^+^ T cells (yellow), CD8^+^ T cells (red) and GrB^+^ cells (green). The scale bars depict 50 µm. **(C)** The frequencies of different immune cell subpopulations in BM and MS are shown with boxplots. Moreover, the spatial distribution of TIL showed a heterogeneous localization in **(D)** BM and **(E)** MS. Significant differences are marked with asterisk (* <0.05; *** > 0.0001). All T cell subsets (green) and CD34^+^ cells (red) including myeloid blasts (asterisk) and endothelial cells (arrow) are shown. Of note, the blasts in MS are frequently negative for CD34. The scale bars depict 30 µm. **(F)** The minimal spatial distance of CD3^+^ to CD3^+^CD8^+^, as well as their localization to every other cell was analyzed and presented with box plots. Significant differences are marked with asterisk (* <0.05; *** > 0.0001). **(G)** Comparison of the frequency of different immune cell subpopulations in the TME of MS (n=45) depending on the HLA-I HC expression (HLA-I HC high H score >150) analyzed by IHC are shown with box plots.

Since the immune cell infiltration of the TME from BM and paired MS might differ, the frequencies and the spatial distribution of CD3^+^CD8^-^ T cells, CD3^+^CD8^+^ T cells, CD3^+^FoxP3^+^ regulatory T cells (Treg) and CD3^-^MUM1^+^ B cells/plasma cells, CD3^+^GrB^+^ T cells were analyzed in 38 paired BMB and MS samples. As representatively shown in **Figures 3A, B**, significant differences in the composition of the immune cell subpopulations and their localization were demonstrated between BMB and MS. In general, all analyzed immune cell subsets showed a lower mean frequency in MS cases. This was accompanied by lower numbers of GrB^+^ cells in MS suggesting an impaired T cell activity (**Figure 3C**). Analysis of the spatial distribution revealed a distinct pattern of immune cell infiltration in MS compared to BMB (**Figures 3D, E**). In BM, a diffuse infiltration and distribution of TIL was detected, while TIL were predominantly located in the periphery of the tumor tissue and in the proximity of blood vessels in most MS representing an immune cell excluded “cold” TME (32/45). Of note, MS cases with a “hot” TME characterized by high TIL numbers within the tumor formation were found in 3/4 isolated MS cases lacking BM pathology. The mean minimal distance of CD3^+^ and CD3^+^CD8^+^ T cells in the MS was higher when compared to the BM (6.38 µm versus 10.01 µm), but exhibited a high variability ranging from 2.31 - 92.23 µm (**Figure 3F**). Since the expression of immune-relevant molecules could be influenced by the immune cell repertoire and vice versa (26, 27), the interrelationship between the HLA-I APM component expression and the local immune cell infiltration was analyzed in MS. While the correlation of the total numbers of all TIL subsets analyzed with HLA-I HC expression demonstrated no significant difference, HLA-I^high^ cases (H score >150) had a higher frequency of CD3^+^CD8^+^ T cells as well as higher numbers of GrB^+^ T cells (**Figure 3G**).

### Impact of the altered immune cell composition and HLA-I APM expression profile on the patients’ survival

**Figure 4:**
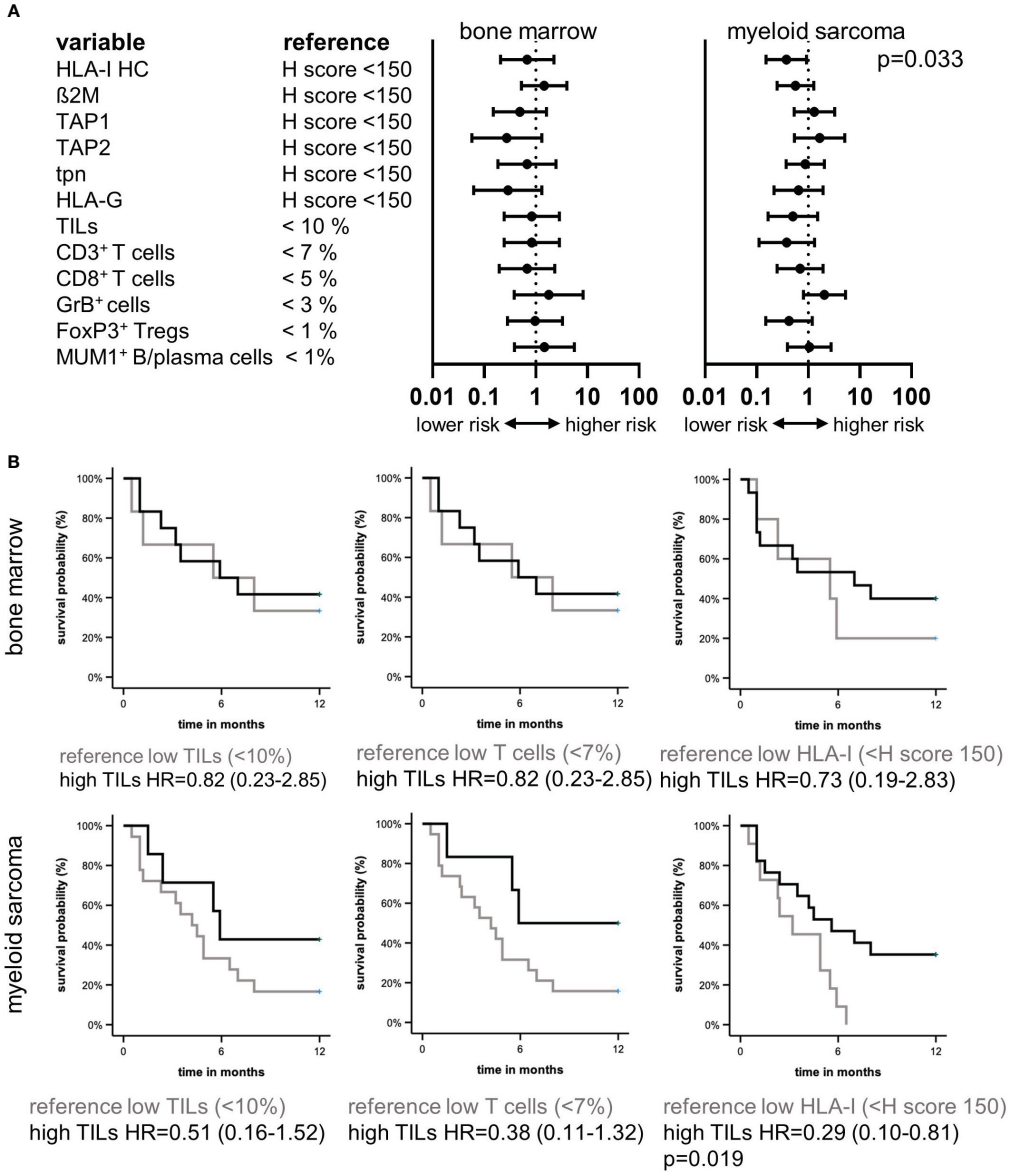
Prognostic relevance of the tumor microenvironment and HLA-I HC in myeloid sarcoma. Forest plots **(A)** of univariate cox regression analysis of the prognostic value of different immune variables in the TME of BMB and MS of 24 patients demonstrated HLA-I HC expression as a prognostic factor in MS. **(B)** Kaplan Meier curvesdepict the survival benefit in MS patients with higher TIL numbers, higher T cell numbers and higher HLA-I HC expression levels.

Based on the interrelation between immune-relevant molecules and the TME composition, the clinical relevance of the HLA-I APM component expression and immune cell infiltration was determined in the MS patients. The average OS of the 24 MS patients with available outcome (follow up time of up to 25 months) was 7.8 months. Forrest plot depiction of univariate cox regression demonstrated a significant influence of HLA-I expression in MS, but not in the corresponding BM. Moreover, higher HLA-I and ß_2_M expression levels accompanied by increased TIL numbers correlated tendentially with a better patients’ outcome (**Figure 4A**), which is also underlined by Kaplan Meier estimators for TIL, T cell numbers and HLA-I HC (**Figure 4B**).

### Comparison of the gene expression pattern of HLA-I^high^ versus HLA-I^low^ MS cases

**Figure 5:**
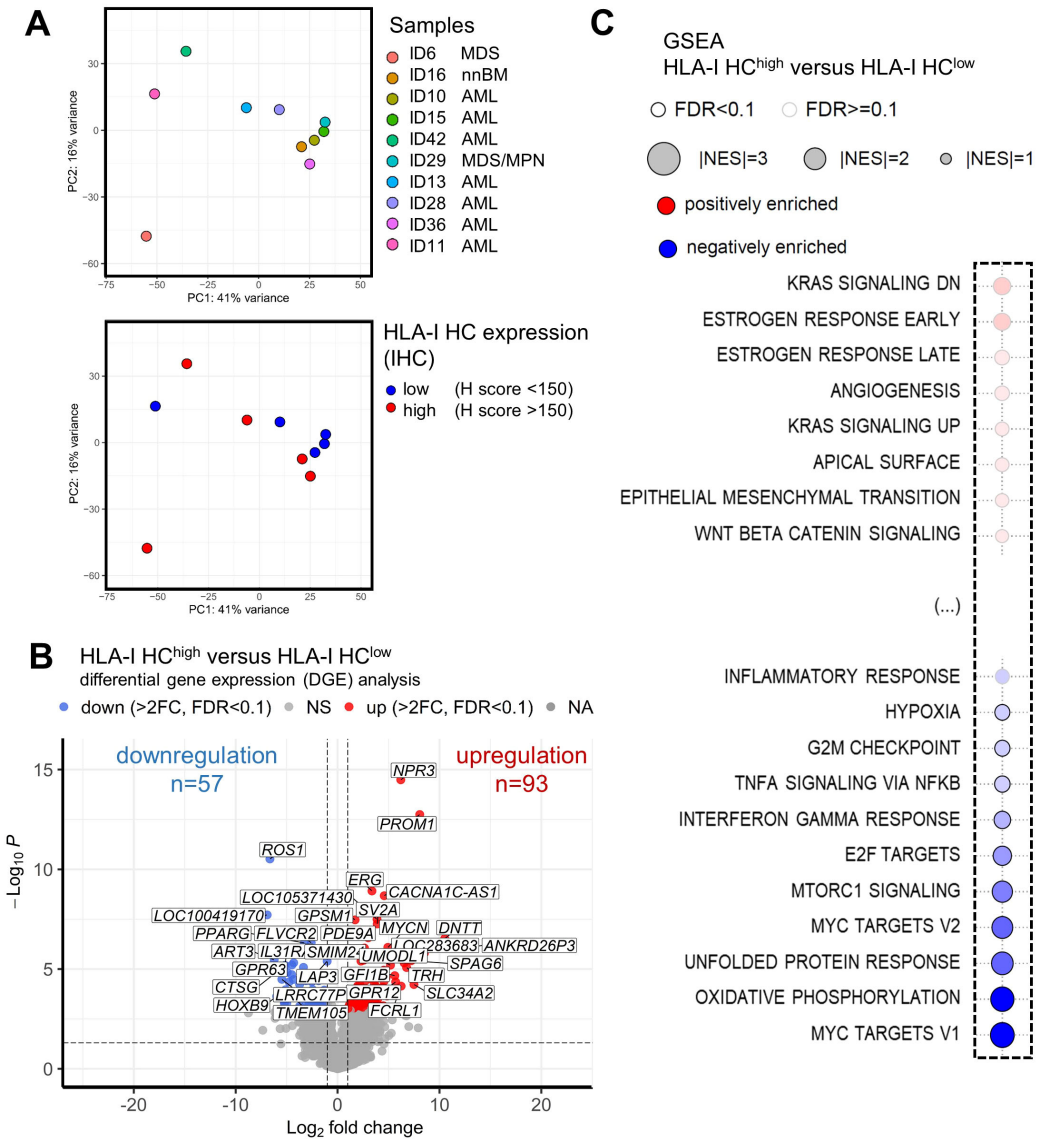
Association of HLA-I expression and gene expression in myeloid sarcoma. RNAseq analysis was performed on 10 samples with preserved (n=5) and reduced (n=5) HLA-I expression. Principal component analysis (PCA) is shown with **(A)** a legend of the different colored dots of the respective samples and their underlying BM findings given on the right side of the plot. A second PCA shows the HLA-I HC expression of these samples analyzed by IHC. Blue dots mark samples with reduced HLA-I HC expression, while red dots mark samples with preserved HLA-I HC expression. **(B)** Results of differential gene expression (DGE) analysis of HLA-I HC^high^ versus HLA-I HC^low^ samples are shown in a volcano plot. Significantly upregulated genes are highlighted in red and significantly downregulated genes are depicted with blue dots. **(C)** Gene set enrichment analysis (GSEA) of MS samples with HLA-I HC^high^ versus HLA-I HC^low^ are depicted with a bubble plot. Non-affected pathways are shown in **Supplementary Figure S1**. Significant differentially regulated pathways are marked with a black line around the bubble and the colors (red – positive enrichment; blue negative enrichment) and the size of the bubble (power of the difference) are depicted in the legend above the bubble plot. The influenced pathways are shown on the left side of the bubble plot.

To get insights into the underlying cause of the better clinical outcome of patients with HLA-I^high^ tumors, the transcriptome of 5 MS cases with high/preserved (HLA-I^high^) and 5 MS cases with reduced HLA-I (HLA-I^low^) expression was determined by RNAseq analyses. Principal component analysis (PCA) revealed that 7/10 MS samples were grouped together. The variation showed neither an association with the underlying BM findings nor with the HLA-I HC expression (**Figure 5A**). As shown in a volcano plot, DGE analysis revealed 93 significantly upregulated and 57 significantly downregulated genes (fold change >2, p<0.05) in HLA-I^high^ versus HLA-I^low^ samples, respectively (**Figure 5B**). The 10 most upregulated genes were involved in immune signaling metabolism and cell differentiation and include e.g. *DNTT*, *PROM1*, and *FCRL1*, while the 10 most significantly downregulated genes in HLA-I^high^ cases were transcription factors and genes involved in immune or cell signaling and/or exhibit enzymatic activity (**Table 3**). Moreover, gene set enrichment analysis (GSEA) (**Figure 5C**, for all pathways see **Supplementary Figure S1**) revealed in samples with preserved HLA-I HC expression a downregulation of inflammatory response genes with significantly lower expression levels of genes involved in TNF-α signaling and interferon-γ response when compared to HLA-I^low^ samples. Moreover, a decreased expression of E2F, the MYC-targets V1 and V2, cell cycle checkpoints and metabolic pathway components was shown in HLA-I^high^ cases. A sustained interferon-γ response was also found in MS samples with high TILs when compared with patients with low TILs in MS formation (**Supplementary Figure S1**). The RNAseq data compared to the GSE103344 dataset showed a down-regulation of HOXB9 and an up-regulation of CTSG in cells that showed mass formation *in vitro* and *in vivo* (**Supplementary Table S2**).

**Table 3:** Top differentially expressed genes.

gene	LOG2FC	p-value	gene name	function
DNTT	10,52	3,0E-07	TdT, DNA nucleotidylexotransferase	immune signaling
PROM1	8,07	1,8E-13	prominin 1	cell differentiation
SLC34A2	7,50	6,0E-05	solute carrier family 34 member 2	metabolism
SPAG6	7,23	3,8E-06	sperm associated antigen 6	immune signaling
TRH	6,78	8,4E-06	thyrotropin releasing hormone	signaling
FCRL1	6,24	7,2E-05	Fc receptor like 1	immune signaling
NPR3	6,21	3,3E-15	natriuretic peptide receptor 3	metabolism
GPR12	5,70	4,4E-05	G protein-coupled receptor 12	cell signaling
GFI1B	5,62	2,1E-05	growth factor independent 1B transcriptional repressor	cell differentiation
EFHC2	4,85	2,2E-04	EF-hand domain containing 2	unknown
HOXB8	-4,95	1,5E-05	homeobox B8	transcription factor
CD5L	-4,98	5,1E-04	CD5 molecule like	immune signaling
IL31RA	-5,16	6,5E-06	interleukin 31 receptor A	immune signaling
FOXD1	-5,21	4,8E-04	forkhead box D1	immune signaling
TRHDE	-5,22	5,2E-04	thyrotropin releasing hormone degrading enzyme	enzyme
CTSG	-5,44	8,5E-06	cathepsin G	immunity
GPR63	-5,60	7,0E-06	G protein-coupled receptor 63	cell signaling
HOXB9	-5,99	2,3E-04	homeobox B9	transcription factor
ART3	-6,23	3,5E-06	ADP-ribosyltransferase 3 (inactive)	cell signaling
ROS1	-6,64	3,0E-11	ROS proto-oncogene 1, receptor tyrosine kinase	cell signaling

Significantly upregulated genes are highlighted in red and significantly downregulated genes are depicted in blue.

## Discussion

In the last two decades, tumor initiation and progression has been shown to be not only influenced by tumor intrinsic factors, like the mutational burden, loss of tumor antigens and HLA-I surface expression and upregulation of ICP, but also by the surrounding TME leading to immune escape, which is one major hallmark of cancer (28). The interrelation of tumor intrinsic and extrinsic factors modulates the disease progression and can be influenced by anti-cancer therapies (29, 30). Moreover, it is known for a long time that the progression of *in-situ* neoplastic lesions in solid tumors are linked with the immunoediting process leading to the development of immune escape variants (31, 32). In the context of hematopoiesis, stem cells are actively integrated in the immune surveillance to safeguard the integrity of the stem cell niche, which significantly differs regarding the immune cell composition of neoplastic BM (22, 33). In addition, inflammasome activation appears to be a crucial mechanism in the pathogenesis of hematopoietic neoplasms and immune evasion strategies have been shown to be involved in disease progression (34). In this study, the influence of immune escape mechanisms within the disease pathogenesis of MS and their clinical significance was investigated. Paired MS and BM analysis revealed a downregulation of HLA-I and APM component expression in MS manifestations in most patients that was associated with an aberrant TME composition and significantly shorter OS. This might explain why MS long-term survivors benefit from the treatment with hypomethylating agents (HMA), which is known to induce tumor antigen expression, upregulate HLA-I molecules as well as APM components thereby enhancing anti-tumor immunity (35–38). Caraffini et al. (21) reported that the loss of RKIP is a frequent event in MS and promotes leukemic tissue infiltration. Interestingly, analysis of the publicly available dataset of their model system demonstrated a significant downregulation of HLA-I and TAP1 thereby confirming our data.

In addition, lymphocytes represent a physiological component of non-neoplastic BM (nnBM) that exhibit usually a diffuse infiltration pattern. This diffuse infiltration pattern was also found for TILs in the BM microenvironment of MN, like MDS, MPN and AML (22, 34), but not in MS, where TILs were predominantly detected in the tumor margin with immune cells accumulating at the interface of neoplastic cells and the surrounding tissue as well as in the proximity to blood vessels as reported in the TME of immune cell excluded carcinoma (23, 39). However, it is unclear, why the diffuse infiltration occurs in neoplastic BM tissues, but not in the TME of MS. It is noteworthy that an immune cell excluded TME has been shown in tumors with low HLA-I expression (26). Furthermore, in many solid tumors the frequency of immune cell subpopulations and their spatial distribution were associated with the patient´s outcomes (39–41). Since this aberrant immune cell excluded pattern was not restricted to MS cases with significantly reduced HLA-I HC expression, we analyzed differences in the transcriptome of MS cases with reduced and preserved HLA-I HC expression, which were associated with significantly downregulated immune signaling pathways suggesting an impaired immunity in both, cases with preserved and reduced HLA-I expression. Moreover, a significant downregulation of E2F and MYC V1 and V2 targets was found in cases with preserved HLA-I HC expression. Both E2F and the MYC oncogene are regulators of immune responses (42), which was linked to a downregulation of HLA-I APM components expression (43, 44) and a T cell poor microenvironment (45) as well as a reduced patients’ survival upon targeted therapy or immune checkpoint inhibitors treatment (46).

The MS manifestation and its TME composition predicted the patient’s survival, while the BM findings showed no association with patient’s survival. In the past it has been clinically shown, that isolated MS cases had a superior survival when compared to MS cases with parallel AML, MDS or MPN (47). In line with these clinical findings, a “hot” or immune cell infiltrated TME defined by high numbers of TILs within the tumor formation was found in most isolated MS cases in our study, which might explain the better patients’ outcome. Based on these data, it could be suggested that the pre-existing MN in the BM have already altered the anti-tumor immunity driving the immune cell excluded TME in many MS cases. Moreover, the use of HMA like azacitidine has been shown to be beneficial for MS patients, that might is related to its immune modulating and activating affects that have been shown before (14, 48).

In conclusion, this study shows a fundamental role of immune escape mechanisms (i) in the initiation of MS disease and (ii) its extramedullary manifestation, which (iii) is associated with an aberrant TME and (iv) the patient´s outcome. However, further studies are urgently needed to identify the underlying intracellular and extracellular mechanisms driving the immune escape in order to develop new treatment strategies for this severe disease with low survival probabilities.

## Data availability statement

The original contributions presented in the study are publicly available. This data can be found here: https://www.ncbi.nlm.nih.gov/geo/query/acc.cgi?acc=GSE273877.

## Ethics statement

The studies involving humans were approved by Ethical Committee of the Medical Faculty, Martin Luther University Halle-Wittenberg, Germany (2017-81 and 2023-196). The studies were conducted in accordance with the local legislation and institutional requirements. Written informed consent for participation in this study was provided by the participants’ legal guardians/next of kin. Written informed consent was obtained from the individual(s) for the publication of any potentially identifiable images or data included in this article.

## Author contributions

MB: Conceptualization, Data curation, Formal Analysis, Investigation, Software, Validation, Visualization, Writing – original draft, Writing – review & editing. AM: Data curation, Investigation, Writing – review & editing. HH: Data curation, Formal Analysis, Investigation, Software, Validation, Visualization, Writing – review & editing. AW: Data curation, Formal Analysis, Investigation, Writing – review & editing. NJ: Data curation, Investigation, Writing – review & editing. HB: Data curation, Investigation, Resources, Writing – review & editing. HA-A: Conceptualization, Resources, Supervision, Writing – review & editing. BS: Conceptualization, Funding acquisition, Resources, Supervision, Writing – original draft, Writing – review & editing. CW: Conceptualization, Project administration, Resources, Supervision, Writing – original draft, Writing – review & editing.

## Supplementary material

Supplementary Figure 1 Gene set enrichment analysis (GSEA) of MS samples with HLA-I HC^high^ versus HLA-I HC^low^ are depicted with a bubble plot. Moreover, also patient samples with high and low TIL numbers were compared.

Supplementary Table 1 Antibodies used.

Supplementary Table 2 Comparison of the HLA-I APM component expression and TIL subset expression of samples from the Pathology Departments in Halle and Leipzig.

Supplementary Table 3 Association of HLA-I APM component expression as well as TIL subsets and the anatomical side of myeloid sarcoma manifestation.

Supplementary Table 4 Association of HLA-I APM component expression and TIL subpopulations and the underlying non-neoplatic or neoplastic BM.

Supplementary Table 5 Differentially gene expression (DGE) of the GSE103344 data set. Genes that were downregulated in our own MS samples are marked with blue, while upregulated genes are highlighted in red. The logarithmic fold change (LOG2FC) of these genes in the GSE103344 data set is given in a separate column with the respective p-values on the right side.
