# Supplementary figures and images for: Association of immune evasion in myeloid sarcomas with disease manifestation and patients’ survival
Source: Front Immunol. 2024 Aug 7;15:1396187. doi: 10.3389/fimmu.2024.1396187 (PMC11336574; doi:10.3389/fimmu.2024.1396187)

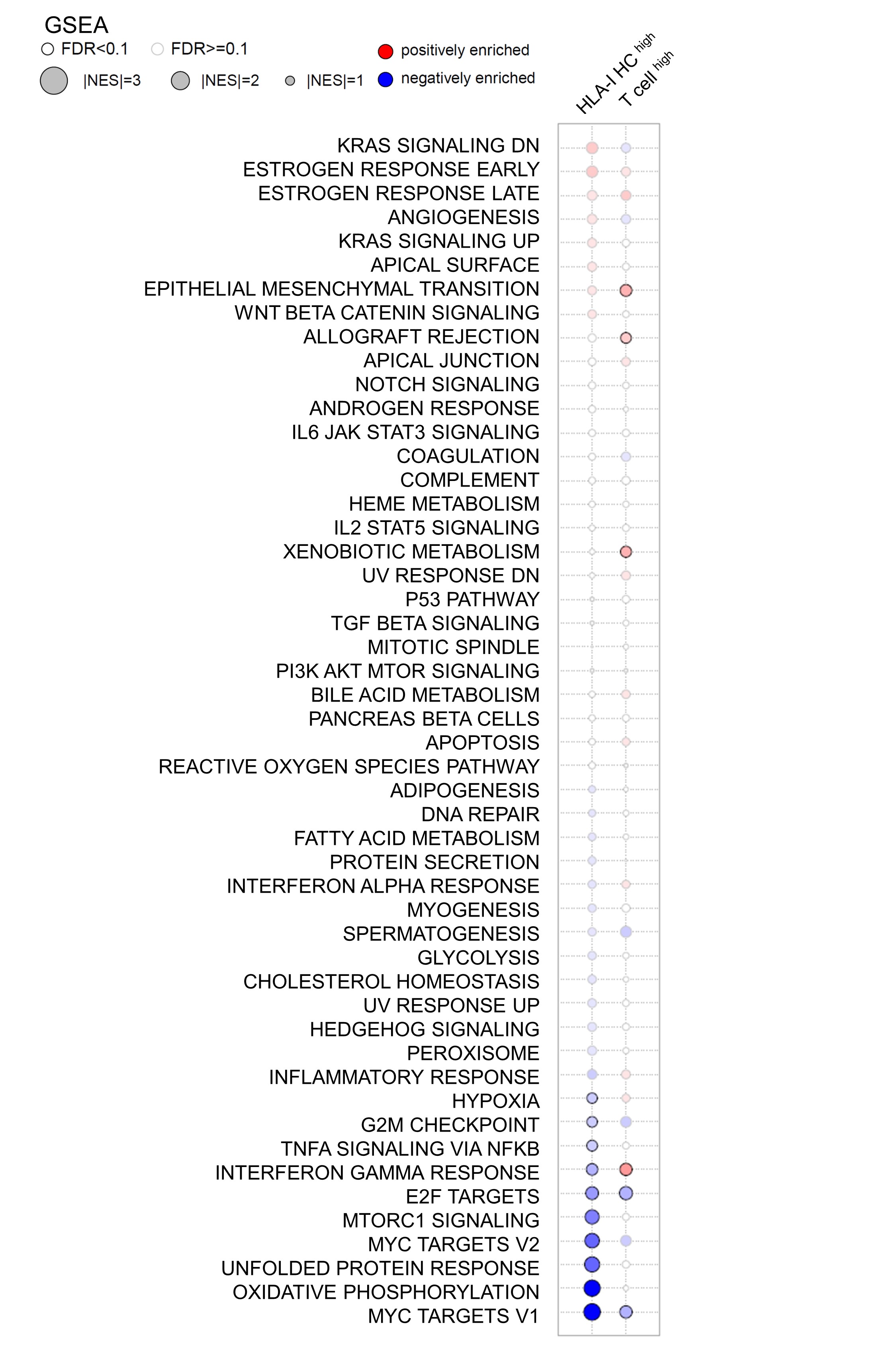

Supplement: Supplementary Figure 1 — Gene set enrichment analysis (GSEA) of MS samples with HLA-I HChigh versus HLA-I HClow are depicted with a bubble plot. Moreover, also patient samples with high and low TIL numbers were compared. [file Image_1.jpeg]
