# Supplementary material for: Association of immune evasion in myeloid sarcomas with disease manifestation and patients’ survival
Source: Front Immunol. 2024 Aug 7;15:1396187. doi: 10.3389/fimmu.2024.1396187 (PMC11336574; doi:10.3389/fimmu.2024.1396187)
Supplement: Supplementary Table 1 — Antibodies used. [file Table_1.docx]

**Supplementary Table S1: Antibodies used.**

| **antibody** | **clone** | **supplier** |
| --- | --- | --- |
| ß_2_M | B2M/961 | Abcam, UK |
| CD3 | SP7 | Labvision, Germany |
| CD8 | SP16 | Abcam, UK |
| CD34 | QBend | Thermo Fisher Scientific, USA |
| CD68 | PG-M1 | Dako, USA |
| CD117 | CD117 | Dako, USA |
| FoxP3 | 236A/E7 | Abcam, UK |
| granzyme B | 262A-14 | Cell marque |
| HLA-G | 4H84 | Abcam, UK |
| lysozyme | EP134 | Epitomics, USA |
| HLA-I HC | HC10 | Thermo Scientific, MA, USA |
| myeloperoxidase | A0398 | Dako, USA |
| MUM1 | MUM1p | Dako, USA |
| TAP1 | ab13516 | Abcam, UK |
| TAP2 | ab180611 | Abcam, UK |
| tpn | ab288565 | Abcam, UK |
