# Supplementary material for: Association of immune evasion in myeloid sarcomas with disease manifestation and patients’ survival
Source: Front Immunol. 2024 Aug 7;15:1396187. doi: 10.3389/fimmu.2024.1396187 (PMC11336574; doi:10.3389/fimmu.2024.1396187)
Supplement: Supplementary Table 2 — Comparison of the HLA-I APM component expression and TIL subset expression of samples from the Pathology Departments in Halle and Leipzig. [file Table_2.docx]

**Supplementary Table S2:** Comparison of the HLA-I APM component expression and TIL subset expression of samples from the Pathology Departments in Halle and Leipzig.

|  |  | **Halle (n=29)** | | | | **Leipzig**  **(n=16)** | | | | | $\boldsymbol{x}$**^2^** | |
| --- | --- | --- | --- | --- | --- | --- | --- | --- | --- | --- | --- | --- |
| **Variable** |  | **min** |  | **max** | **mean** | **min** |  | **max** | **mean** | **p-value** | |  |
| **HLA-I HC** | **H score** | 20 | - | 300 | 163 | 0 | - | 240 | 159 | 0.266 | |  |
| **ß2M** | **H score** | 10 | - | 300 | 156 | 0 | - | 200 | 99.4 | 0.244 | |  |
| **TAP1** | **H score** | 10 | - | 250 | 122 | 70 | - | 250 | 169 | 0.196 | |  |
| **TAP2** | **H score** | 0 | - | 300 | 113 | 0 | - | 300 | 122 | 0.429 | |  |
| **tpn** | **H score** | 0 | - | 200 | 108 | 30 | - | 220 | 128 | 0.169 | |  |
| **HLA-G** | **H score** | 0 | - | 250 | 34,6 | 0 | - | 200 | 29.1 | 0.447 | |  |
| **TILs** | **%** | 0.2 | - | 36.1 | 9 | 1.3 | - | 40,3 | 10,5 | 0.426 | |  |
| **T cells** | **%** | 0.1 | - | 17.4 | 3,46 | 0.2 | - | 20.1 | 4.6 | 0.426 | |  |
| **CD8^+^ T cells** | **%** | 0 | - | 2.2 | 0,36 | 0.1 | - | 4.6 | 0.8 | 0.190 | |  |
| **FoxP3^+^ Tregs** | **%** | 0 | - | 17.4 | 0,89 | 0 | - | 0,8 | 0.1 | 0.496 | |  |
| **GrB^+^ cells** | **%** | 0 | - | 6.2 | 0,71 | 0 | - | 2,5 | 0.4 | 0.486 | |  |
| **MUM1^+^ B/plasma cells** | **%** | 0 | - | 19.9 | 1,36 | 0 | - | 2.9 | 0.7 | 0.240 | |  |
| **T cell distance** | **µm** | 5.6 | - | 937 | 352 | 24.3 | - | 628 | 168 | 0.418 | |  |
