# Supplementary material for: Association of immune evasion in myeloid sarcomas with disease manifestation and patients’ survival
Source: Front Immunol. 2024 Aug 7;15:1396187. doi: 10.3389/fimmu.2024.1396187 (PMC11336574; doi:10.3389/fimmu.2024.1396187)
Supplement: Supplementary Table 3 — Association of HLA-I APM component expression as well as TIL subsets and the anatomical side of myeloid sarcoma manifestation. [file Table_3.docx]

**Supplementary Table S3:** Association of HLA-I APM component expression as well as TIL subsets and the anatomical side of myeloid sarcoma manifestation.

|  |  | | **skin (n=28)** | | | | **non-immune privileged organ (n=9)** | | | | **immune-privileged organ (n=7)** | | | | $\boldsymbol{x}$**^2^** |
| --- | --- | --- | --- | --- | --- | --- | --- | --- | --- | --- | --- | --- | --- | --- | --- |
| **Variable** |  | | **min** |  | **Max** | **mean** | **min** |  | **max** | **mean** | **min** |  | **max** | **mean** | **p-value** |
| **HLA-I HC** | | H score | 0 | - | 300 | 173.9 | 20 | - | 240 | 137.8 | 90 | - | 200 | 142.9 | 0.718 |
| **ß2M** | | H score | 0 | - | 300 | 152.5 | 10 | - | 200 | 104.4 | 10 | - | 220 | 107.1 | 0.378 |
| **TAP1** | | H score | 10 | - | 250 | 150.4 | 30 | - | 200 | 123.3 | 30 | - | 200 | 112.9 | 0.517 |
| **TAP2** | | H score | 10 | - | 300 | 135.5 | 0 | - | 200 | 90.0 | 0 | - | 300 | 96.7 | 0.530 |
| **tpn** | | H score | 0 | - | 220 | 129.3 | 0 | - | 200 | 66.7 | 50 | - | 200 | 120.0 | 0.641 |
| **HLA-G** | | H score | 0 | - | 200 | 32.0 | 0 | - | 250 | 41.1 | 0 | - | 100 | 24.3 | 0.291 |
| **TILs** | | % | 0.2 | - | 11.2 | 11.2 | 0.2 | - | 22.5 | 5.9 | 0.4 | - | 18,8 | 8.5 | 0.417 |
| **T cells** | | % | 0.1 | - | 4.8 | 4.8 | 0,1 | - | 3.2 | 1.4 | 0.2 | - | 8.9 | 4.1 | 0.471 |
| **CD8^+^ T cells** | | % | 0.0 | - | 0.5 | 0.5 | 0 | - | 2.2 | 0.8 | 0.0 | - | 0.9 | 0.2 | 0.387 |
| **FoxP3^+^ Tregs** | | % | 0.0 | - | 0.8 | 0.8 | 0 | - | 1.7 | 0.3 | 0.0 | - | 1.1 | 0.3 | 0.346 |
| **GrB^+^** ^cells^ | | % | 0.0 | - | 0.9 | 0.9 | 0 | - | 0.3 | 0.1 | 0.0 | - | 1.2 | 0.3 | 0.402 |
| **MUM1^+^ B/ plasma cells** | | % | 0.0 | - | 0.7 | 0.7 | 0 | - | 19.9 | 3.0 | 0.0 | - | 0.2 | 0.1 | 0.374 |
| **T cell**  **distance** | | µm | 24.3 | - | 937.2 | 264,5 | 5.6 | - | 546.4 | 173.2 | 46.5 | - | 783.5 | 389.7 | 0.408 |
