# Supplementary material for: Association of immune evasion in myeloid sarcomas with disease manifestation and patients’ survival
Source: Front Immunol. 2024 Aug 7;15:1396187. doi: 10.3389/fimmu.2024.1396187 (PMC11336574; doi:10.3389/fimmu.2024.1396187)
Supplement: Supplementary Table 4 — Association of HLA-I APM component expression and TIL subpopulations and the underlying non-neoplatic or neoplastic BM. [file Table_4.docx]

|  |  | **non-neoplastic BM (n=4)** | | | | **MPN (n=11)** | | | | **MDS and MDS/MPN (n=5)** | | | | **AML (n=24)** | | | | $\boldsymbol{x}$**^2^** | |
| --- | --- | --- | --- | --- | --- | --- | --- | --- | --- | --- | --- | --- | --- | --- | --- | --- | --- | --- | --- |
| **Variable** |  | **Min** |  | **Max** | **Mean** | **Min** |  | **Max** | **Mean** | **Min** |  | **Max** | **Mean** | **Min** |  | **Max** | **Mean** | **p-value** |  |
| **HLA-I HC** | H score | 140 | - | 200 | 167.5 | 0 | - | 300 | 163.6 | 40 | - | 240 | 158.0 | 20 | - | 300 | 160.4 | 0.663 |  |
| **ß2M** | H score | 10 | - | 180 | 107.5 | 0 | - | 250 | 132.7 | 110 | - | 130 | 120.0 | 10 | - | 300 | 144.6 | 0.348 |  |
| **TAP1** | H score | 140 | - | 200 | 175.0 | 70 | - | 240 | 137.3 | 100 | - | 200 | 138.0 | 10 | - | 250 | 133.8 | 0.137 |  |
| **TAP2** | H score | 70 | - | 300 | 163.3 | 0 | - | 150 | 64.0 | 120 | - | 120 | 120.0 | 0 | - | 300 | 120.0 | 0.910 |  |
| **Tpn** | H score | 40 | - | 200 | 122.5 | 0 | - | 200 | 65.5 | 110 | - | 220 | 164.0 | 0 | - | 200 | 126.3 | 0.087 |  |
| **HLA-G** | H score | 0 | - | 250 | 117.5 | 0 | - | 120 | 18.6 | 0,0 | - | 0.0 | 0.0 | 0 | - | 200 | 31.7 | **0.033** |  |
| **TILs** | % | 0.4 | - | 22.5 | 13.6 | 0.2 | - | 25.4 | 10.4 | 1.4 | - | 5.0 | 3.1 | 0.2 | - | 40.3 | 10.1 | 0.407 |  |
| **T cells** | % | 0.2 | - | 8.9 | 3.4 | 0.1 | - | 11.0 | 4.3 | 0.2 | - | 1.8 | 1.1 | 0.0 | - | 20.1 | 4.5 | 0.407 |  |
| **CD8^+^ T cells** | % | 0.0 | - | 1.0 | 0.3 | 0.0 | - | 4.6 | 1.0 | 0.0 | - | 0.7 | 0.3 | 0.0 | - | 2.2 | 0.4 | 0.178 |  |
| **FoxP3^+^ Tregs** | % | 0.0 | - | 0.6 | 0.2 | 0.0 | - | 0.9 | 0.2 | 0.0 | - | 1.7 | 0.4 | 0.0 | - | 17.4 | 0.9 | 0.831 |  |
| **GrB^+^ cells** | % | 0.0 | - | 0.1 | 0.0 | 0.0 | - | 3.2 | 0.9 | 0.0 | - | 1.6 | 0.4 | 0.0 | - | 6.2 | 0.6 | 0.540 |  |
| **MUM1^+^ B/ plasma cells** | % | 0.0 | - | 19.9 | 6.6 | 0.0 | - | 2.6 | 0.8 | 0.0 | - | 1.3 | 0.5 | 0.0 | - | 7.0 | 0.6 | 0.518 |  |
| **T cell**  **Distance** | µm | 86.1 | - | 783.5 | 505.4 | 5.6 | - | 534.2 | 130.7 | 65.1 | - | 546.4 | 182.7 | 40.8 | - | 937.2 | 315.6 | 0.397 |  |

**Supplementary Table S4:** Association of HLA-I APM component expression and TIL subpopulations and the underlying non-neoplatic or neoplastic BM
