# Supplementary material for: Association of immune evasion in myeloid sarcomas with disease manifestation and patients’ survival
Source: Front Immunol. 2024 Aug 7;15:1396187. doi: 10.3389/fimmu.2024.1396187 (PMC11336574; doi:10.3389/fimmu.2024.1396187)
Supplement: Supplementary Table 5 — Differentially gene expression (DGE) of the GSE103344 data set. Genes that were downregulated in our own MS samples are marked with blue, while upregulated genes are highlighted in red. The logarithmic fold change (LOG2FC) of these genes in the GSE103344 data set is given in a separate column with the respective p-values on the right side. [file Table_5.docx]

**Supplementary Table S5:** Differentially gene expression (DGE) of the GSE103344 data set. Genes that were downregulated in our own MS samples are marked with blue, while upregulated genes are highlighted in red. The logarithmic fold change (LOG2FC) of these genes in the GSE103344 data set is given in a separate column with the respective p-values on the right side.

| **GSE103344 data set** | | |
| --- | --- | --- |
| **gene** | **LOG2FC** | **p-value** |
| DNTT | -0,25 | 0.916 |
| PROM1 | -0,16 | 0.557 |
| SLC34A2 | -0,22 | 0.811 |
| SPAG6 | -0,23 | 0.936 |
| TRH | 0,28 | 0.567 |
| FCRL1 | -0,33 | 0.915 |
| NPR3 | 0,00 | 0.946 |
| GPR12 | 0,41 | 0.575 |
| GFI1B | 0,21 | 0.969 |
| EFHC2 | 0,01 | 0.957 |
| HOXB8 | -0,41 | 0.732 |
| CD5L | -0,59 | 0.124 |
| IL31RA | -0,29 | 0.959 |
| FOXD1 | -0,64 | 0.198 |
| TRHDE | -0,31 | 0.916 |
| CTSG | 0,51 | **0.020** |
| GPR63 | 0,00 | 0.936 |
| HOXB9 | -1,92 | **0.001** |
| ART3 | -0,25 | 0.936 |
| ROS1 | -0,23 | 0.818 |
